# Elucidating the Urothelial-Dependent and -Independent Mechanisms Involved in the Mouse Bladder Contractility Alterations by Acute Methylglyoxal Exposure

**DOI:** 10.3390/biomedicines14051017

**Published:** 2026-04-30

**Authors:** Akila Lara Oliveira, Matheus Leite Medeiros, Vivian Fuguhara, Idam Hermawan, Hiroaki Shimokawa, Masato Tsutsui, Gilberto De Nucci, Edson Antunes

**Affiliations:** 1 Department of Pharmacology, University of Campinas (UNICAMP), Campinas 13084-971, Brazil; 2 Department of Pharmacology, Graduate School of Medicine, University of the Ryukyus, Okinawa 903-0215, Japan

**Keywords:** advanced glycation end products, glyoxalase, Y27632, RAGE antagonist peptide, HC-030031, HC-067047

## Abstract

**Background/Objectives**: Methylglyoxal (MGO) and subsequent activation of advanced glycation end products (AGEs)–RAGE receptor signaling has been implicated in the complications of diabetes mellitus (DM), such as bladder dysfunction. Chronic treatment with MGO leads to bladder overactivity, but the effects of acute MGO exposure have not yet been evaluated. **Methods**: In this study, we used female wild-type, endothelial nitric oxide (eNOS) knockout (eNOS^−/−^), and triple (neuronal/endothelial/inducible) NOS^−/−^ mice to investigate the effects of incubation of MGO (10 to 300 µM) on bladder contractions induced by carbachol and electrical field stimulation (EFS). We also analyzed the activity of the MGO detoxification enzyme glyoxalase 1 (Glo1). **Results**: Incubation with MGO at 10 and 30 µM in urothelium-intact preparations produced marked detrusor hypercontractility to both carbachol and EFS that was abolished by urothelium removal. Detrusor hypercontractility was associated with the generation of reactive oxygen species (ROS), RAGE activation, Rho kinase sensitization, and activation of TRPA1 and TRPV4 channels. At higher concentrations (100 and 300 µM), MGO did not significantly affect the detrusor contractility to carbachol and EFS, but L-NAME pretreatment restored the hypercontractile state by MGO. Likewise, in bladder strips obtained from eNOS^−/−^ or triple NOS^−/−^ mice, MGO exposure (300 µM) significantly enhanced carbachol and EFS-induced contractions, indicating a major role for nitric oxide (NO) counteracting the hypercontractility. No concentration of MGO altered Glo1 activity in bladder tissues. **Conclusions**: In conclusion, progressive MGO accumulation may account for the transition from the initial hyperactive phase to the subsequent hypoactive decompensated phase of diabetic bladder dysfunction.

## 1. Introduction

Methylglyoxal (MGO) as a glucose co-product is a highly reactive dicarbonyl compound that readily interacts with proteins, leading to the formation of advanced glycation end products (AGEs) and consequent loss of protein function [1]. Endogenous glycation, responsible for the formation of AGEs, results from non-enzymatic reactions between reducing sugars and dicarbonyl compounds, such as MGO, with proteins [2]. In short-lived proteins with rapid turnover, only initial glycation products, such as Amadori products, are formed. The formation of AGEs requires prolonged exposure, making long-lived proteins more susceptible [3]. MGO is primarily detoxified by the glyoxalase system, which comprises two enzymes: glyoxalase I (Glo1; lactoylglutathione methylglyoxal lyase) and glyoxalase II (Glo2; hydroxyacylglutathione hydrolase), responsible for converting MGO into D-lactate [4]. Once formed, AGEs bind to their membrane-anchored receptor (RAGE), a member of the immunoglobulin superfamily of cell surface receptors that recognize endogenous ligands [5]. AGE–RAGE interaction promotes the generation of reactive oxygen species (ROS) and drives cellular dysfunction [5,6].

Understanding the effects of MGO is particularly relevant in the context of hyperglycemia associated with diabetes mellitus (DM), a condition in which circulating MGO levels are markedly elevated [7,8]. MGO has been implicated in multiple diabetes-related complications, including diabetic nephropathy, endothelial dysfunction, post-infarction cardiac remodeling, and impaired insulin signaling [9,10,11,12]. In addition, diabetic bladder dysfunction (DBD) is a highly prevalent condition, affecting over 50% of patients [13,14], which may be related to high MGO levels [15,16].

In previous studies, we investigated the impact of chronic MGO exposure on bladder function using a model in which animals received MGO (0.5%) in drinking water for 12 weeks [17,18,19]. Chronic intake of MGO led to bladder overactivity and detrusor hypercontractility, accompanied by increased AGE formation, activation of the RAGE–ROS signaling axis, upregulation of L-type calcium channels, and Rho-kinase-mediated muscle sensitization [18]. Notably, we demonstrated the critical involvement of the urothelium in modulating detrusor contractile responses, as well as the upregulation and activation of the transient receptor potential ankyrin 1 (TRPA1) channel, whose blockade effectively mitigated MGO-induced bladder hyperactivity [19]. However, these findings reflect a chronic exposure model in which multiple structural and molecular alterations coexist, including elevated serum levels of MGO and AGEs, bladder hypertrophy, urothelial and detrusor thickening, increased collagen content, and reduced Glo1 activity [17,18,19]. This complex pathological context hinders the identification of the direct effects of MGO, making it necessary to distinguish between primary actions of the compound and those mediated through downstream signaling via AGEs, and Glo1.

We hypothesized that MGO alone alters detrusor contractility through direct effects. To address this, the present study aimed to investigate the direct effects of MGO on detrusor contractility, independently of AGE formation or Glo1 activation. We performed in vitro functional assays using isolated bladder strips incubated with varying concentrations of MGO (10, 30, 100, and 300 µM) for 30 min. Additionally, we explored the involvement of pharmacological targets previously implicated in detrusor hypercontractility in the chronic model, including the use of the Rho kinase inhibitor Y27632, polyethyleneglycol-superoxide dismutase (PEG-SOD), the RAGE antagonist peptide (RAP), the TRPA1 antagonist HC-030031, and the TRPV4 antagonist HC-067047. We also showed the involvement of nitric oxide (NO) release in detrusor contractility in bladders exposed to MGO by using the NO synthesis inhibitor L-NAME, as well as tissues obtained from endothelial nitric oxide (eNOS) knockout (eNOS^−/−^) and triple (neuronal/endothelial/inducible) NOS^−/−^ mice. Enzymatic activity of Glo1 was also assessed following incubation in the bladder strips to determine its potential contribution to the observed contractile changes, enabling us to isolate the direct actions of MGO on detrusor function.

## 2. Materials and Methods

Drugs.

The following drugs were used: carbachol (CAS Number 51-83-2, St. Louis, Missouri, Sigma-Aldrich, USA), Y27632 (Catalog No. Y050; Sigma-Aldrich, St. Louis, MO, USA), HC-030031 (Catalog No. H4415; Sigma-Aldrich, St. Louis, MO, USA), HC-067047 (Catalog No. SML0143; Sigma-Aldrich, St. Louis, MO, USA), RAP (Catalog No. 553031; Sigma-Aldrich, St. Louis, MO, USA), methylglyoxal solution (Catalog No. M0252; Sigma-Aldrich, St. Louis, MO, USA), PEG-SOD (Catalog No. S9549; Sigma-Aldrich, St. Louis, MO, USA), and N^ω^-nitro-L-arginine methyl ester hydrochloride (L-NAME; 483125-M; Sigma-Aldrich, St. Louis, MO, USA).

Animals.

Animals were obtained from CEMIB (Multidisciplinary Center for Biological Research in the Field of Laboratory Animal Science—UNICAMP, Sao Paulo, Brazil). Seventeen-week-old female C57BL/6 mice (25 ± 0.30 g) were used in the final experiment. Animals were maintained under standard bioterium conditions and housed in polypropylene cages (41 × 34 × 18 cm; ten animals per cage) placed in a ventilated rack system. Environmental conditions were controlled, with constant humidity (55 ± 5%), temperature (24 ± 1 °C), and a 12 h light/dark cycle. Animals had free access to standard chow and filtered water. Environmental enrichment was provided using ANIMAL PRO^®^ Ambient Trio, Sao Paulo, Sao Paulo, Brazil, and The Andersons^®^ Bed-r’Nest, Maumee, OH, USA (Animal Pro; https://www.animalpro.com.br/enriquecimento, accessed on 26 October 2025). Animals were kept under these conditions until they reached 17 weeks of age, at which point the experimental procedures were conducted. The choice of sex and age was based on previous studies [17,18,19]. This study employed females to investigate whether the pathways previously identified in a chronic MGO ingestion model (12 weeks) would also be observed under acute MGO incubation. All experimental procedures were approved by the Ethics Committee on Animal Use of UNICAMP (CEUA-UNICAMP; Protocol No. 6497-1/2024). This study was conducted in accordance with the Brazilian Guide for the Care and Use of Animals for Scientific and Educational Purposes (CONCEA) [20], the PREPARE guidelines [21], and the ARRIVE guidelines [22].

Female triple nitric oxide synthase knockout (n/i/eNOS^−/−^) and eNOS^−/−^ (B6.129P2-*Nos3^tm1Unc^*/J) mice, aged 15 weeks and on a C57BL/6 genetic background, were used in the study. The original colony was generously provided by Dr. M. Tsutsui (School of Medicine, University of Occupational and Environmental Health, Kitakyushu, Japan), and eNOS^−/−^ (B6.129P2-*Nos3^tm1Unc^*/J) mice were obtained from Jackson Laboratory (Bar Harbor, ME, USA). Local breeding was established and maintained by CEMIB/UNICAMP. The phenotypic and physiological features of triple NOS knockout (n/i/eNOS^−/−^) mice have been described in an earlier study [23]. All experimental procedures were approved by the Ethics Committee on Animal Use of UNICAMP (CEUA-UNICAMP; Protocol No. 6447-1/2024).

In vitro incubation with MGO and functional tests in intact and denuded bladders.

At 17 weeks of age, animals were anesthetized using isoflurane at a concentration greater than 5%, followed by cervical dislocation to confirm euthanasia. The bladder was immediately excised and carefully divided into two strips: one with intact urothelium and the other denuded. For preparation of the denuded bladder, the urothelium along with the lamina propria was gently removed using fine-tip forceps, as previously described [18]. The strips were then mounted in 10 mL organ baths filled with Krebs–Henseleit solution (117 mM NaCl, 4.7 mM KCl, 2.5 mM CaCl_2_, 1.2 mM MgSO_4_, 1.2 mM KH_2_PO_4_, 25 mM NaHCO_3_, and 5.5 mM glucose; pH 7.4), at 37 °C, continuously aerated with 95% O_2_ and 5% CO_2_. Tissues were allowed to equilibrate for 45 min, with tension adjusted to 5 mN every 15 min, and the solution was renewed at each interval. After stabilization, one strip was incubated with vehicle (saline), and the other with MGO (10, 30, 100, or 300 µM), for 30 min. Isometric contractions were recorded using a PowerLab system (ADInstruments Inc., Sydney, Australia). Following incubation, electrical field stimulation (EFS) was applied via two platinum ring electrodes connected to a stimulator (Grass Technologies, West Warwick, RI, USA). EFS was delivered at 80 V, with a 1 ms pulse width, in 10 s trains at frequencies ranging from 1 to 32 Hz, with 2 min intervals between stimulations. Cumulative concentration–response curves to the muscarinic agonist carbachol (0.1 nM–100 µM; Sigma-Aldrich, St. Louis, MO, USA) were also generated [24]. Contractile responses were normalized to tissue weight and expressed as mN/mg.

Evaluation of pharmacological agents in intact bladder strips

In a separate set of experiments, intact bladder strips were preincubated (30 min) or not with MGO in the presence of either vehicle (saline or 0.001% dimethylsulfoxide (DMSO)) or the following pharmacological agents: Y27632 (1 µM), PEG-SOD (87 IU/mL), RAP (10 µM), HC-030031 (10 µM), HC-067047 (10 µM), or L-NAME (100 µM). Following incubation, contractile responses were evaluated using EFS and cumulative concentration–response curves to carbachol (0.1 nM–100 μM).

Glyoxalase 1 activity in intact bladder strips

Bladders were divided into two longitudinal strips and allocated into groups according to the MGO concentration (vehicle, 10, 30, 100, or 300 µM). Each strip was placed in 10 mL of Krebs–Henseleit solution, maintained at 37 °C and continuously aerated with a gas mixture of 95% O_2_ and 5% CO_2_. After a 10 min equilibration period, tissues were incubated for 40 min with the assigned MGO concentration. Following incubation, each strip was individually frozen by group for subsequent analysis of Glo1 activity. Tissues were homogenized in 350 µL of sodium phosphate buffer (pH 7.0) and centrifuged at 2000× *g* for 30 min at 4 °C. The supernatant was collected and kept on ice. Glo1 activity was measured in duplicate using the Glyoxalase I Activity Assay Kit (Catalog No. MAK114, Sigma-Aldrich, USA), following the manufacturer’s instructions. Results were normalized to total protein content, quantified in triplicate using the DC™ Protein Assay Kit II (Catalog No. 5000112, Bio-Rad Laboratories, Inc., Hercules, CA, USA) [17].

Statistical analysis

All statistical analyses were performed using GraphPad Prism software, version 8 (GraphPad Software, Inc., San Diego, CA, USA). The normality of data distribution was assessed using the Shapiro–Wilk test. Comparisons between two groups were conducted using the unpaired Student’s *t*-test. For comparisons among three or more groups, one-way analysis of variance (ANOVA) was applied, followed by Dunnett’s multiple comparisons test. Results are expressed as mean ± standard error of the mean (SEM), or standard deviation (SD), as specified in the descriptive statistics. Differences were considered statistically significant when *p* < 0.05. Concentration–response data for carbachol were fitted to a nonlinear regression model using a variable-slope sigmoidal dose–response equation of the form: *E* = *E_max_*/[1 + (10*^c^*/10*^x^*)*^n^*] + *F*, where *E* is the effect above baseline, *E_max_* is the maximum response, *c* is the logarithm of the pEC_50_ (the concentration producing 50% of the maximal response), *x* is the logarithm of the drug concentration, *n* is the Hill slope (curve-fitting parameter), and *F* is the response in the absence of the drug. Effect sizes were calculated to determine the magnitude of differences between groups. For all comparisons, Hedges’ g (a version of Cohen’s *d* corrected for small sample sizes) and its 95% confidence intervals (CIs) were computed using the Psychometrica online calculator [25]. For parameters derived from nonlinear regression (pEC_50_), the standard error (SE) of the fit was converted to standard deviation (SD) based on the sample size (*n*) to ensure accurate effect size estimation. Effect size magnitudes were interpreted as small (0.2), medium (0.5), or large (0.8 or greater).

## 3. Results

### 3.1. Effect of MGO on Carbachol- and EFS-Induced Bladder Contractions in Intact Urothelial Preparations

**Figure 1:**
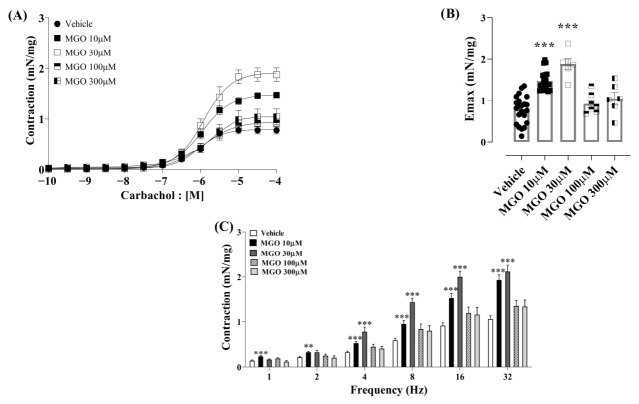
Effect of incubation with vehicle (saline) or methylglyoxal (MGO) at concentrations of 10, 30, 100, and 300 µM on bladders with intact urothelium. Vehicle or MGO was incubated for 30 min. Panel (**A**) shows the concentration–response curve to carbachol (10^−10^ to 10^−4^ M) and panel (**B**) shows the maximal contractile responses (Emax) to this agonist. Panel (**C**) shows the electrical field stimulation-induced contractions (EFS, 1–32 Hz). Data are presented as mean ± SEM. ** *p* < 0.01 and *** *p* < 0.001 compared to vehicle-incubated strips. One-way ANOVA followed by Dunnett’s multiple comparisons test versus vehicle groups.

Incubation of isolated bladders with MGO (10 to 300 µM) did not significantly alter baseline contraction in any concentration used. However, prior incubation with lower MGO concentrations (10 and 30 µM) significantly enhanced (*p* < 0.001) the maximal contractions (*E_max_*) parameter with a large effect size to MGO at 10 µM (Cohen’s *d* = 2.43; 95% CI [1.62, 3.24]) and 30 µM (Cohen’s *d* = 3.35; 95% CI [2.09, 4.61]) compared to vehicle-treated groups (Figure 1A,B and Table 1). No statistically significant difference in pEC_50_ was observed between vehicle and MGO groups, with a small effect size for this parameter (Table 1). Likewise, at 10 and 30 µM, MGO exposure significantly increased EFS-induced (1–32 Hz) bladder contractions (*p* < 0.001; Figure 1C).

In contrast, higher MGO concentrations (100 and 300 µM) did not modify the carbachol (Emax and pEC_50_; Table 1) and EFS responses compared with the vehicle group (Figure 1A–C).

### 3.2. Effect of MGO on Carbachol- and EFS-Induced Bladder Contractions in Denuded Urothelial Preparations

**Figure 2:**
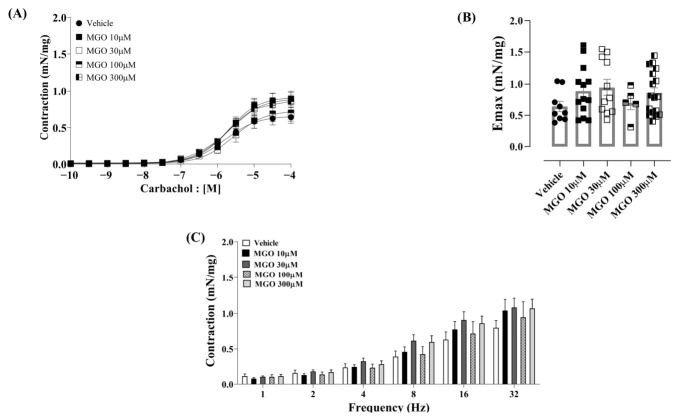
Effect of incubation with vehicle (saline) or methylglyoxal (MGO) at concentrations of 10, 30, 100, and 300 µM on urothelium-denuded bladders. Vehicle or MGO was incubated for 30 min. Panels (**A**,**B**) show the contractile responses to carbachol (10^−10^ to 10^−4^ M). Panel (**C**) shows the electrical field stimulation-induced contractions (EFS, 1–32 Hz). Data are presented as mean ± SEM. One-way ANOVA followed by Dunnett’s multiple comparisons test versus vehicle groups.

Subsequently, we investigated whether the effects of MGO were dependent on the presence of the urothelium; therefore, bladders were incubated with the same MGO concentrations (10 to 300 µM) in preparations where the urothelium was mechanically removed, leaving the detrusor muscle layer intact. The results are shown in Figure 2. At all concentrations tested, MGO did not significantly alter the contractile responses to carbachol, as evidenced by the unaltered parameters of Emax or pEC_50_, a finding corroborated by predominantly low-to-moderate effect sizes (Figure 2A,B and Table 2). The EFS-induced contractions also remained unchanged by MGO in urothelium-denuded preparations (Figure 2C). These findings indicate that enhanced contractile responses observed with the lower MGO concentrations (Figure 1A–C) are dependent on the presence of the urothelium.

### 3.3. Effects of PEG-SOD, Y27632, and RAP on the Enhancement by MGO of Carbachol- and EFS-Induced Bladder Contractions

**Figure 3:**
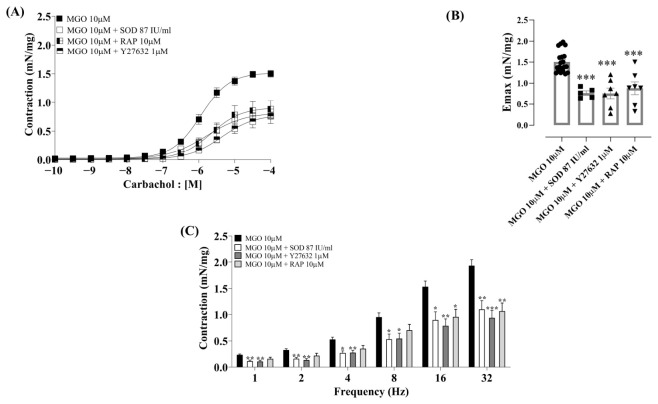
Effect of co-incubation of methylglyoxal (MGO, 10 µM) with superoxide dismutase (PEG-SOD; 87 IU/mL), the Rho kinase inhibitor Y27632 (1 μM), or the RAGE antagonist peptide (RAP; 10 μM) on contractile responses to carbachol and electrical field stimulation (EFS). Each compound was incubated for 30 min. Panels (**A**,**B**) show the concentration–response curves to carbachol (10^−10^ to 10^−4^ M), and panel (**C**) shows EFS-induced (1–32 Hz) responses. Data are presented as mean ± SEM. * *p* < 0.05, ** *p* < 0.01 and *** *p* < 0.001 compared to the MGO 10 μM group. One-way ANOVA followed by Dunnett’s multiple comparisons test versus the MGO group.

We next evaluated the underlying mechanisms mediating the enhancement by MGO (10 µM) of carbachol- and EFS-induced contractions in urothelium-intact bladder strips, giving particular attention to the involvement of oxidative stress, Rho kinase activity, and RAGE signaling, using the antioxidant enzyme PEG-SOD, the selective Rho kinase inhibitor Y27632, or the RAGE antagonist peptide (RAP). Figure 3A–C show preincubation with PEG-SOD (87 IU/mL), Y27632 (1 µM), and RAP (10 µM).

PEG-SOD abrogated the MGO-induced increase in Emax to carbachol (*p* < 0.001; Cohen’s *d* = −3.16; 95% CI [−4.49, −1.83]). For the pEC50 parameter, the difference did not reach statistical significance (*p* = 0.52), although a large effect size was observed (d = −0.88; 95% CI [−1.9, 0.13]). The increased EFS-induced contractions by MGO were also significantly abrogated by PEG-SOD (*p* < 0.01; Figure 3C).

Preincubation with Y27632 (1 µM) significantly reduced both Emax to carbachol with a large effect size (*p* < 0.0001, Cohen’s *d* = −2.71; 95% CI [−3.85, −1.58]) and pEC_50_ (*p* < 0.0001, Cohen’s *d* = −2.49; 95% CI [−3.59, −1.39]; Figure 3A,B and Table 3). The increased EFS-induced responses by MGO were also significantly reduced by Y27632 (*p* < 0.01; Figure 3C).

RAP (10 µM) markedly reduced the potentiation by MGO (10 µM) on carbachol-induced contractions (*p* < 0.001, Cohen’s *d* = −2.09; 95% CI [−3.31, −1.05]). Regarding the pEC50 parameter, the analysis revealed a large effect size (d = 1.2; 95% CI [−2.13, −0.28]), although the *p*-value was borderline (*p* = 0.05; Figure 3A,B and Table 3). Regarding the EFS-induced contractions, RAP significantly reduced the contractions at 16 and 32 Hz only (*p* < 0.05; Figure 3C).

### 3.4. Effects of TRP Channel Antagonists on MGO-Mediated Enhancement of Carbachol- and EFS-Induced Bladder Contractions

**Figure 4:**
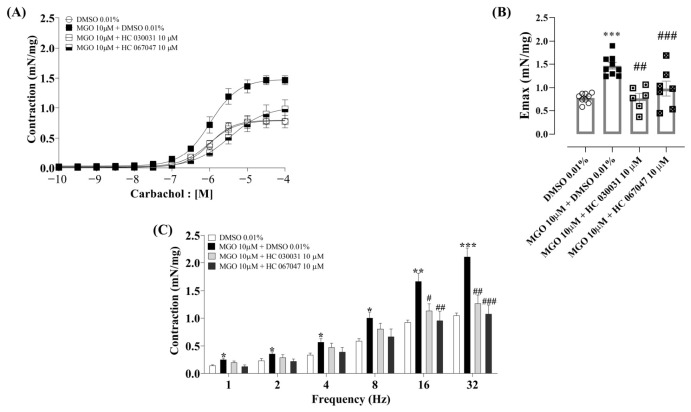
Effect of the selective TRPA1 antagonist HC-030031 and the selective TRPV4 antagonist HC-067047 on the enhancement by MGO of the contractile responses to carbachol and electrical field stimulation (EFS). Each compound was incubated for 30 min. Panels (**A**,**B**) show the contractile responses to carbachol (10^−10^ to 10^−4^ M). Panel (**C**) shows EFS-induced (1–32 Hz) responses. Data are expressed as mean ± SEM. * *p* < 0.05, ** *p* < 0.01, and *** *p* < 0.001 compared to the DMSO 0.01% vehicle. One-way ANOVA followed by Dunnett’s multiple comparisons test versus vehicle group. ^#^ *p* < 0.05, ^##^ *p* < 0.01, and ^###^ *p* < 0.001 compared to the MGO 10 µM + DMSO 0.01% group. One-way ANOVA followed by Dunnett’s multiple comparisons test versus MGO group.

We investigated the effect of TRP channel antagonism by incubating bladders exposed to MGO (10 µM) with the selective antagonists HC-030031 (TRPA1; 10 µM) and HC-067047 (TRPV4; 10 µM). DMSO (0.01%) was used as a vehicle control. The results are shown in Figure 4A–C.

MGO significantly increased the contractile response to carbachol (*p* < 0.05), with the magnitude of this effect considered extremely high (Cohen’s *d* = −3.16; 95% CI [−4.49, −1.83]) compared to vehicle. No significant alterations in the pEC_50_ values were observed (*p* = 0.99), which was corroborated by a very small effect size (d = −0.14; 95% CI [−1,06, 0,78]; Figure 4A,B and Table 4). Co-incubation of MGO with either HC-030031 or HC-067047 reversed the contractile response to carbachol (*p* < 0.0001), with large effect sizes for Emax (HC-030031: d = −3.05; HC-067047: d = −1.51). There were no alterations in pEC_50_ (*p* = 0.99), and the analysis indicated a small effect size (Table 4).

MGO (10 µM) also significantly increased the contractile response to EFS (1–32 Hz; *p* < 0.05) compared to the DMSO group. Co-incubation with either HC-030031 or HC-067047 markedly reduced EFS-induced responses, particularly at the frequencies of 16 and 32 Hz (*p* < 0.05; Figure 4C).

### 3.5. Involvement of Nitric Oxide (NO) as a Compensatory Mediator Released by High MGO Concentrations

**Figure 5:**
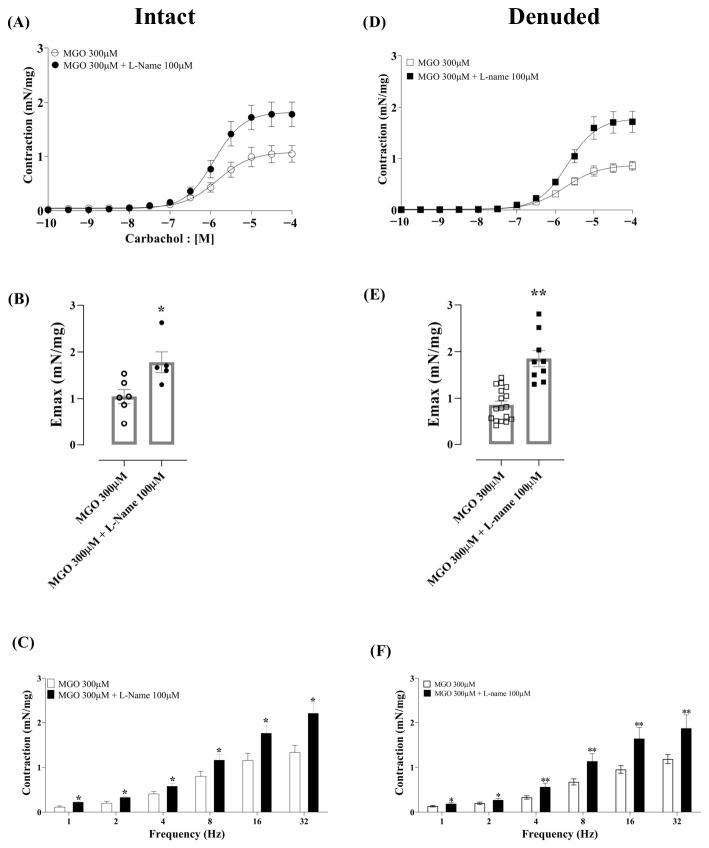
Effect of co-incubation of MGO (300 μM) with the nitric oxide (NO) synthesis inhibitor L-NAME (100 μM) on bladder contractility in urothelium-intact and -denuded bladders. Each compound was incubated for 30 min. Panels (**A**,**B**) show the contractile responses to carbachol (10^−10^ to 10^−4^ M), and panel (**C**) shows responses to electrical field stimulation (EFS, 1–32 Hz) in intact bladder. Panels (**D**,**E**) show the contractile responses to carbachol (0.1–100 μM), and panel (**F**) shows responses to electrical field stimulation (EFS, 1–32 Hz) in the denuded bladder. Data are presented as mean ± SEM. * *p* < 0.05 and ** *p* < 0.01 compared to the MGO 300 μM group in intact or denuded bladders (unpaired Student’s *t*-test).

Given that MGO at high concentrations (100 and 300 µM) failed to increase carbachol- and EFS-induced contractions, we hypothesized that NO could be a compensatory substance released by MGO that counteracts the hypercontractility machinery. To test this, intact and denuded bladders were incubated with MGO (300 µM) in the absence and the presence of L-NAME (100 µM). The results are shown in Figure 5A–F.

Co-incubation of MGO (300 µM) with L-NAME in both intact (Figure 5A–C) and denuded bladder strips (Figure 5D–F) significantly increased the Emax responses to carbachol, with large effect sizes for intact (*p* = 0.02; Cohen’s *d* = −1.71; 95% CI [0.32, 3.09]) and denuded strips (*p* < 0.0001; Cohen’s *d* = 2.47; 95% CI [1.42, 3.52]). No differences were observed in pEC_50_, with small effect sizes for intact (d = −0.32) and denuded preparations (d = −0.27; Table 5). Co-incubation of MGO (300 µM) with L-NAME in both intact and denuded bladder strips (Figure 5A–F) significantly increased the contractile responses to EFS (*p* < 0.05), reaching levels comparable to those observed with the lower MGO concentrations (Figure 5A).

**Figure 6:**
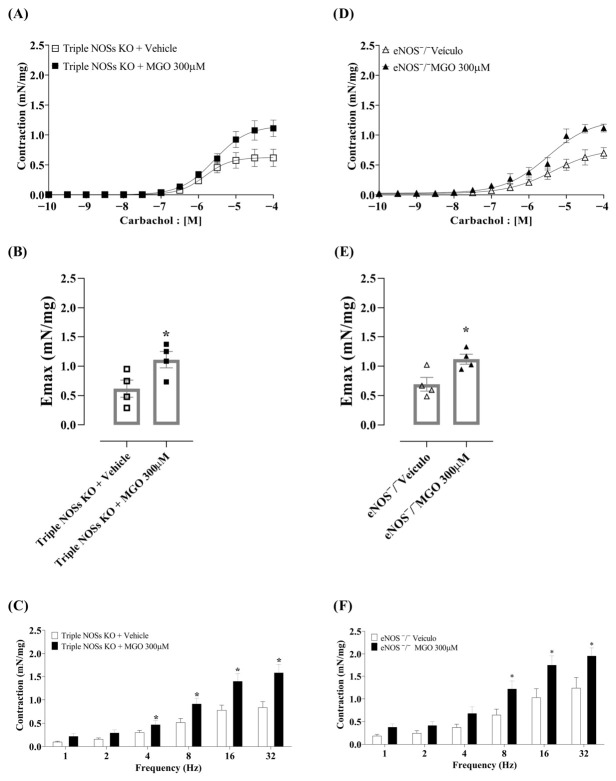
Effect of incubation with MGO (300 μM) in intact bladders from triple nitric oxide synthase (NOS; n/i/eNOS^−/−^) and eNOS (eNOS^−/−^) knockout mice. Each compound was incubated for 30 min. Panels (**A**–**C**) show the contractile responses to carbachol (0.1–100 μM) and electrical field stimulation (EFS, 1–32 Hz) in n/i/eNOS^−/−^ mice. Panels (**D**–**F**) show the contractile responses to carbachol (10^−10^ to 10^−4^ M) and to EFS (1–32 Hz) in eNOS^−/−^ mice. Data are presented as mean ± SEM. * *p* < 0.05 compared to vehicle group (unpaired Student’s *t*-test).

We then incubated intact bladders from triple NOS knockout mice (n/i/eNOS^−/−^) with MGO (300 µM). The results are shown in Figure 6A–C. In bladders from n/i/eNOS^−/−^ mice, MGO significantly (*p* = 0.04) increased contractile responses to carbachol with a large effect size (Cohen’s *d* = 1.75; 95% CI [0.12, 3.38]). Regarding the pEC_50_ values to carbachol, although it did not reach statistical significance (*p* = 0.19), a substantial reduction in terms of effect size measure was observed (d = −1.03; Table 6). The wide confidence interval (95% CI [−2.51, 0.44]) reflects the inherent variability of the pEC_50_ parameter given the small sample size (*n* = 4). MGO also significantly increased contractile responses to EFS (*p* < 0.05).

Sequentially, we incubated the bladders of eNOS^−/−^ mice with MGO (300 µM), and the results are presented in Figure 6D–F. Similar to the triple NOS^−/−^ mice, incubation with MGO caused a significant increase in the Emax responses to carbachol (*p* = 0.02; Cohen’s *d* = 2.12; 95% CI [0.38, 3.85]), whereas pEC_50_ values did not differ between groups (*p* = 0.70; d = 0.28; Table 7). Incubation with MGO also significantly increased EFS-induced contractions (*p* < 0.05). These results reinforce that high concentrations of MGO induce NO release, which counteracts the hypercontractile machinery observed at lower MGO concentrations.

### 3.6. Effect of MGO on Glo1 Activity in Intact Bladder

To evaluate whether Glo1 activity was altered by MGO incubation, bladders were incubated with different concentrations of MGO (10 to 300 µM), and Glo1 activity was subsequently assessed. The results are presented in Table 8. Incubation with MGO did not produce significant changes in Glo1 activity in the bladder in any concentration.

## 4. Discussion

Chronic MGO accumulation has been implicated in vascular complications of DM through mechanisms largely dependent on irreversible cross-linking of AGEs formation and RAGE activation [7]. Chronic MGO accumulation has also been implicated in the urological complications of DM, such as bladder dysfunction [16]. Accordingly, long-term oral MGO exposure in mice leads to activation of AGEs–RAGE signaling in bladder tissues, resulting in bladder overactivity and ex vivo detrusor hypercontractility accompanied by structural (bladder hypertrophy and increased collagen content) and molecular alterations (reduced Glo1 activity) [17,19]. Besides activating this classical downstream signaling via cross-linked AGEs and Glo1, MGO is also described to exert direct actions that may be independent of this pathway activation [26,27]. Therefore, in this study, we sought to determine whether MGO could directly affect bladder contractility and the mechanisms involved. To the best of our knowledge, this is the first study examining the acute effects of MGO on bladder contractility. DBD is a multifactorial condition involving alterations in the detrusor, urothelium, and autonomic nerves, manifesting with a wide spectrum of urinary tract symptoms. A temporal progression is observed, beginning with an initial compensatory phase followed by a decompensated phase of bladder dysfunction [28]. Clinically, this is reflected in symptoms ranging from overactive bladder to voiding dysfunction and urinary retention [14].

In healthy individuals, the estimated production rate of MGO in tissues is approximately 125 µM, primarily derived from the degradation of triose phosphates, and MGO levels remain in the submicromolar range due to efficient glyoxalase activity [29,30]. Plasma MGO concentrations in healthy individuals are typically in the low micromolar to submicromolar range (~0.05–0.3 µM) [31,32,33,34]. On the other hand, plasma and urine from pre-diabetic and diabetic individuals, as well as from obese subjects, exhibit concentrations ranging from 233 nM to 400 µM [35,36,37,38,39,40,41]. Accordingly, the concentrations selected in the present study were intended to approximate those reported in these individual groups, thereby reflecting pathophysiological-relevant levels.

Our data showed that 30 min incubation with MGO differentially affects the bladder contractility according to the concentration employed, that is, lower concentrations (10 and 30 µM) significantly enhanced bladder contractions due to activation of multiple mechanisms related to increased calcium sensitivity and contractile function, whereas higher MGO concentrations (100 and 300 µM) failed to elevate bladder contractions as a result of NO release likely to mask the hypercontractility state of the detrusor smooth muscle. These findings suggest that progressive MGO accumulation may drive the transition from the initial hyperactive phase to the subsequent hypoactive phase of DBD.

During the voiding phase, parasympathetic tone predominates [23]. Postganglionic parasympathetic axons of the pelvic nerve release acetylcholine (ACh), which induces bladder contraction by stimulating muscarinic M3 receptors in the detrusor [23]. Activation of muscarinic receptors is also linked to increased expression and activity of L-type voltage-operated calcium channels in bladder smooth muscle [36]. Based on this mechanism, in the present study, we investigated detrusor contractility mediated by the selective muscarinic receptor agonist carbachol and by EFS, which primarily induces acetylcholine release.

The bladder is a hollow organ lined by a specialized wall known as the urothelium, which is in contact with the lumen [37]. Classified as a stratified epithelium, it consists of multiple layers, with the superficial layer composed of umbrella cells [37]. The morphophysiology of the urothelium differs from other epithelia due to morphological transitions that vary according to the urinary fluid received from the ureters [38]. Its functions include forming an impermeable barrier to ions, solutes, and pathogens, amplifying sensory signals, and interacting with nerve fibers, smooth muscle, blood vessels, and interstitial cells [38]. The urothelium responds to a variety of chemical, mechanical, and thermal stimuli [37,38]. Therefore, we next explored whether urothelium played a role in producing hypercontractility at the lower MGO concentrations. We found that urothelium removal abolished the ability of MGO to enhance the carbachol- and EFS-induced contractions, placing urothelium as the primary site of action of MGO to prime the contractile machinery, leading to hypercontractility. Previously, we demonstrated that 12-week treatment with MGO led to bladder hypercontractility as a result of increased AGEs formation and RAGE expression, leading to excessive ROS production, Rho kinase dysregulation, and increased calcium machinery [18]. Other studies have also shown that acute MGO exposure increases oxidative stress [39,40,41], leading to upregulation of the Rho kinase system and calcium sensitivity in rat fibroblasts and vascular smooth muscle [42,43,44,45].

To investigate whether ROS formation and Rho kinase activation were involved in the urothelium-dependent hypercontractility produced by MGO exposure (10 µM), co-incubation with the antioxidant enzyme SOD or the Rho kinase inhibitor Y27632 was performed. Each treatment fully inhibited bladder hypercontractility by MGO preincubation, strongly suggesting that sequential excess ROS formation and Rho kinase activation in urothelium ultimately lead to an enhanced calcium signal in detrusor smooth muscle in a similar fashion with the chronic MGO treatment [18].

Activation of RAGE has been mainly determined in studies employing subacute and chronic treatments with MGO [46,47] in conditions of irreversible cross-linking AGEs formation. Increased RAGE expression by MGO exposure has been mostly identified in isolated and cultured cells [48,49]. Few studies have explored RAGE localization and function in bladder tissues, in urothelium and detrusor smooth muscle [50,51]. In our study, incubation of intact bladders with RAP (RAGE antagonist peptide) reduced the hypercontractility to 10 µM MGO, indicating that 30 min exposure to MGO can already activate RAGE, triggering its downstream signaling involving ROS formation and Rho kinase activation.

Nevertheless, it remains to be elucidated whether RAGE activation by short-term incubation with MGO involves the formation of irreversible AGE cross-links [1,2]. Since AGEs are widely recognized as RAGE activators [6], in the acute model investigated here, it appears unlikely that short-term MGO exposure generates mature, cross-linked AGEs, given the limited incubation time. Such cross-linked AGEs are typically observed under chronic diabetic conditions [4]. Thus, the findings of this study seem to reflect the immediate and direct effects of MGO, or the formation of early glycation intermediates, which are driven by rapid, reversible, or early-stage modifications affecting detrusor contractility. This mechanism differs from the long-term effects observed under chronic treatment, which involve cross-linked AGE formation, tissue remodeling, increased collagen deposition, and tissue stiffening [17,18,19].

In the sensory regulation of the bladder, mechanisms such as the micturition threshold, perception of filling, and bladder pain may involve TRP activation [52]. The TRP superfamily (TRPA, TRPV, and TRPM) comprises a wide variety of cation channels with diverse physiological and pathological functions [53]. When activated, these channels induce changes in membrane excitability and regulate extracellular calcium influx. TRPA1 and TRPV4 channels are present in the bladder, acting as sensors of stretch and/or chemical irritation, and are found both in primary afferent neurons and in the urothelium [52,53]. Previously, we demonstrated that chronic MGO treatment increased TRPA1 expression, manifesting as an overactive bladder phenotype [19]. Consistent with this, MGO has been reported to activate TRPA1, thereby enhancing calcium influx [54,55,56]. Moreover, MGO promoted pain through spinal sensitization of TRPA1, leading to hyperalgesia in db/db mice [57]. Importantly, MGO selectively activated TRPA1 in dorsal root ganglion neurons and HEK293t cells, thereby stimulating Aδ and C nociceptors in vitro [58]. In the present study, we investigated whether MGO-induced hypercontractility involves TRPA1 activation, and further examined TRPV4, a channel gaining attention in overactive bladder dysfunction. TRPV4 is abundantly expressed in the urothelium and also present in the subepithelium, afferent neurons, and detrusor smooth muscle, where it can directly regulate urothelial barrier function and detrusor contractility [59,60]. Here, we demonstrate that acute incubation with MGO in the bladder activated both TRPA1 and TRPV4, as evidenced by our findings that the antagonists HC030031 (TRPA1) and HC067047 (TRPV4) reduced MGO-induced hypercontractility (10 µM) in response to carbachol and EFS. These data suggest that MGO can activate more than one TRP channel, highlighting that molecular mechanisms may be more complex and tissue dependent.

Subsequently, we investigated the mechanism(s) implicated in the loss of hypercontractility at high MGO concentrations (100 and 300 µM). Based on the hypothesis that this phenomenon could be related to the concomitant release of relaxing factors, such as NO, we used the NO synthesis inhibitor L-NAME and bladder strips from eNOS^−/−^ and triple (neuronal/endothelial/inducible) NOS^−/−^ mice. NO, as a relaxing mediator of bladder smooth muscle, has been identified in the urothelium and detrusor smooth muscle [61]. In urothelium-intact bladder strips exposed to 300 µM of MGO, treatment with L-NAME increased hypercontractility to carbachol and EFS, an effect also reproduced in urothelium-denuded preparations. We next evaluated the effects of MGO incubation in bladder strips from animals lacking NO production due to gene knockout of NOS isoforms, namely, eNOS^−/−^ and triple NOS^−/−^ mice. In both groups, incubation with MGO (300 µM) resulted in detrusor hypercontractility in response to carbachol and EFS, contrasting with bladder responses from wild-type animals in the absence of L-NAME. Therefore, our findings suggest that MGO exposure is associated with eNOS-derived NO release in the detrusor smooth muscle layer, which may act to counterbalance the hypercontractile response. Thus, mechanisms related to calcium sensitization and muscle contraction appear to predominate at lower MGO concentrations (10 and 30 µM), whereas higher MGO concentrations (100 and 300 µM) may recruit compensatory pathways that attenuate hypercontractility. Although this hypothesis is biologically plausible and supported by functional assays, the present study did not directly assess whether high concentrations of MGO induce NO production, requiring additional studies to directly quantify NO levels to confirm this mechanism. Moreover, although NO-mediated compensation represents a plausible explanation for the observed biphasic response, alternative mechanisms, such as receptor desensitization or cytotoxic effects at higher MGO concentrations, cannot be excluded and should be addressed in future investigations.

Finally, to determine whether the effects of MGO incubation involve its degradation, Glo1 activity was evaluated in bladders exposed to MGO. Glo1, the enzyme responsible for MGO degradation, is a limiting factor for the accumulation of MGO and the formation of AGEs [1]. Glo1 activity is significantly reduced in bladder tissues from chronic MGO treatment that is consistent with the presence of true dicarbonyl stress [17,19]. However, in the present study, incubation of bladders with increasing concentrations of MGO (10 to 300 µM) did not alter enzymatic activity, indicating that the observed changes of detrusor contractility are attributable to the direct presence of MGO.

Although this study was conducted in an ex vivo model, exclusively in female animals, this represents a limitation regarding the generalizability of the findings. Therefore, potential sex-related differences in bladder physiology should be considered when interpreting these findings.

**Figure 7:**
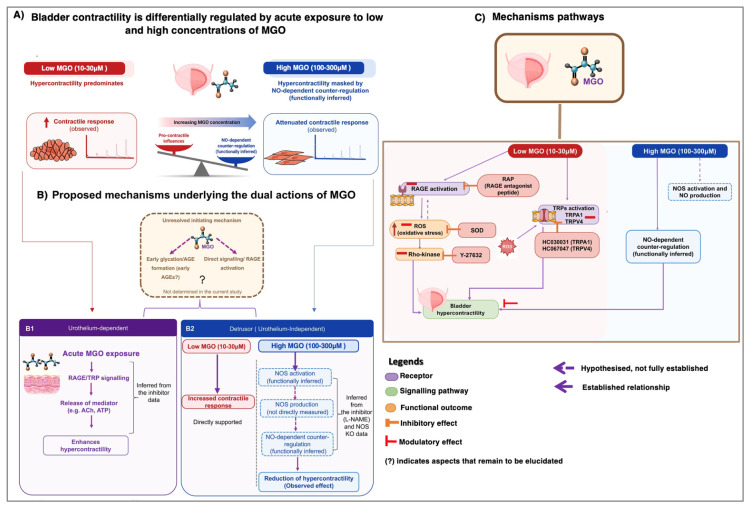
Proposed mechanisms for the acute effects of MGO (10 to 300 µM, 30 min) on the bladders of female mice. (**A**) Acute MGO exposure at 10 and 30 µM produces urothelium-dependent detrusor hypercontractility to carbachol (CCh) and electrical field stimulation (EFS), whereas at higher MGO concentrations (100 and 300 µM), the hypercontractility was masked by NO-dependent counter-regulation (functionally inferred). (**B**) The proposed mechanism underlying the dual action of MGO has not yet been established, particularly regarding whether its effects depend on the formation of AGEs or on MGO per se directly activating RAGE. What is established is that MGO exerts dual actions, both dependent and independent of the urothelium. Panel (**B1**) illustrates the urothelium-dependent action of MGO, whereas panel (**B2**) shows the urothelium-independent action. (**C**) Investigated mechanisms to elucidate the altered detrusor contractile responses to MGO exposure. Considering that the generation of complex AGEs is time dependent, while AGE dimers can form rapidly, our data with RAP (RAGE antagonist peptide) preincubation suggest that acute MGO incubation may trigger AGE-dependent or -independent mechanisms, resulting in RAGE activation, without excluding the possibility that MGO could directly activate RAGE. Increased ROS production and oxidative stress as a consequence of classical RAGE activation enhance detrusor contractility, which is suppressed by SOD preincubation. Increased ROS production in turn activates the Rho kinase signaling, predisposing the detrusor smooth muscle to a hypercontractile state due to calcium sensitization, an effect blocked by preincubation with the Rho kinase inhibitor Y-27632. Another mechanism associated with detrusor hypercontractility due to MGO exposure is the activation of TRPA1 and TRPV4, which can be activated by MGO itself and/or by excess ROS formation. Accordingly, preincubation with HC-030031 (TRPA1 inhibitor) or HC-067047 (TRPV4 inhibitor) mitigated the hypercontractility. Finally, acute MGO exposure at higher concentrations (100 and 300 µM) induces NO release from the detrusor, counteracting the hypercontractile state of the muscle, as evidenced by preincubation with L-NAME or using bladders from endothelial nitric oxide (eNOS) knockout (eNOS^−/−^) and triple (neuronal/endothelial/inducible) NOS^−/−^ mice. This image was produced with the assistance of Servier Medical Art (https://smart.servier.com; accessed on 23 April 2026) licensed by a Creative Commons Attribution 3.0 Unported License.

Overall, our results suggest that progressive accumulation of MGO under diabetic conditions may contribute to the transition from an initial hypercontractile to a subsequent hypocontractile state of the detrusor (a proposed mechanism has been presented in Figure 7). This work, however, represents an initial step toward understanding the direct effects of MGO per se, independent of long-term alterations, such as increased collagen deposition or reduced Glo1 activity. Experiments in diabetic animal models, such as ob/ob and db/db mice, should be conducted to investigate whether there is a threshold for MGO-induced changes in detrusor contractility. These models are particularly relevant, as diabetic animals already exhibit elevated serum MGO levels due to their metabolic state [15,62,63]. Further studies are also required to determine whether the dual effects observed here are also present in models in vivo.

## 5. Conclusions

The present study, supported by in vitro pharmacological assays, demonstrates that acute MGO exposure exerts a dual effect on detrusor contractility, namely, at lower concentrations (10–30 µM), it favors a hypercontractility state associated with increased ROS generation and downstream activation of multiple signaling pathways, including RAGE, Rho kinase, and TRPA1 and TRPV4 channels. At higher concentrations (100–300 µM), MGO promotes NO release, which counterbalances the contractile responses observed with the lower concentrations.

## Figures and Tables

**Table 1:** Values of Emax and potency (pEC_50_) of carbachol-induced contractions in intact bladder strips incubated with different concentrations of MGO.

	Emax (mN/mg)							
Group	*n*	Mean	SD	Comparison	*p*-Value	Cohen’s *d*	CI Lower (95%)	CI Upper (95%)
Vehicle	22	0.77	0.33	_				
MGO 10 μM	19	1.49	0.25	vs. Vehicle	<0.0001	2.43	1.62	3.24
MGO 30 μM	6	1.87	0.32	vs. Vehicle	<0.0001	3.35	2.09	4.61
MGO 100 μM	7	0.92	0.24	vs. Vehicle	0.70	0.48	−0.37	1.34
MGO 300 μM	6	1.04	0.37	vs. Vehicle	0.20	0.47	−0.43	1.38
	**pEC_50_**							
Vehicle	22	6.05	0.28	_				
MGO 10 μM	19	5.95	0.17	vs. Vehicle	0.49	−0.42	−1.05	0.2
MGO 30 μM	6	5.92	0.14	vs. Vehicle	0.61	−0.5	−1.41	0.41
MGO 100 μM	7	5.94	0.26	vs. Vehicle	0.69	−0.39	−1.25	0.45
MGO 300μM	6	5.82	0.31	vs. Vehicle	0.13	−0.8	−1.73	0.12

Data are expressed as mean ± SD. Effect size was calculated as Cohen’s *d*, accompanied by 95% confidence intervals (CIs). One-way ANOVA followed by Dunnett’s multiple comparisons test versus vehicle groups.

**Table 2:** Values of Emax and potency (pEC_50_) of carbachol-induced contractions in denuded bladder strips incubated with different concentrations of MGO.

	Emax (mN/mg)							
Group	*n*	Mean	SD	Comparison	*p*-Value	Cohen’s *d*	CI Lower (95%)	CI Upper (95%)
Vehicle	9	0.64	0.24	_				
MGO 10 μM	13	0.89	0.39	vs. Vehicle	0.31	0.73	−0.13	1.61
MGO 30 μM	11	0.94	0.42	vs. Vehicle	0.19	0.85	−0.06	1.77
MGO 100 μM	5	0.69	0.24	vs. Vehicle	0.99	0.2	−0.88	1.3
MGO 300 μM	17	0.85	0.34	vs. Vehicle	0.39	0.67	−0.15	1.5
	**pEC50**							
Vehicle	9	5.79	0.36	_				
MGO 10 μM	13	5.70	0.32	vs. Vehicle	0.94	−0.26	−1.12	0.58
MGO 30 μM	11	5.72	0.40	vs. Vehicle	0.98	−0.18	−1.06	0.7
MGO 100 μM	5	5.57	0.29	vs. Vehicle	0.63	−0.65	−1.77	0.46
MGO 300 μM	17	5.75	0.37	vs. Vehicle	0.990	−0.1	−0.91	0.69

Data are expressed as mean ± SD. Effect size was calculated as Cohen’s *d*, accompanied by 95% confidence intervals (CIs). One-way ANOVA followed by Dunnett’s multiple comparisons test versus vehicle groups.

**Table 3:** Values of Emax and potency (pEC_50_) of carbachol-induced contractions in intact bladder strips incubated with MGO (10 μM) in the presence or not of PEG-SOD (87 IU/mL), Y27632 (1 μM), or RAP (10 μM).

	Emax (mN/mg)							
Group	*n*	Mean	SD	Comparison	*p*-Value	Cohen’s *d*	CI Lower (95%)	CI Upper (95%)
MGO (untreated)	19	1.49	0.25	_				
MGO + PEG-SOD	5	0.76	0.11	vs. untreated	<0.0001	−3.16	−4.49	−1.83
MGO + Y27632	7	0.75	0.33	vs. untreated	<0.0001	−2.71	−3.85	−1.58
MGO + RAP	7	0.88	0.39	vs. untreated	<0.0001	−2.09	−3.13	−1.05
	**pEC_50_**							
MGO (untreated)	19	5.95	0.17	_				
MGO + PEG-SOD	5	5.78	0.27	vs. untreated	0.52	−0.88	−1.9	0.13
MGO + Y27632	7	5.36	0.37	vs. untreated	<0.0001	−2.49	−3.59	−1.39
MGO + RAP	7	5.65	0.40	vs. untreated	0.05	−1.2	−2.13	−0.28

Data are expressed as mean ± SD. Effect size was calculated as Cohen’s *d*, accompanied by 95% confidence intervals (CIs). One-way ANOVA followed by Dunnett’s multiple comparisons test versus vehicle groups.

**Table 4:** Values of Emax and potency (pEC_50_) of carbachol-induced contractions in intact bladder strips incubated with vehicle (0.01% DMSO) or MGO (10 μM) in the presence or not of HC-030031 (10 μM) or HC-067047 (10 μM).

	Emax (mN/mg)							
Group	*n*	Mean	SD	Comparison	*p*-Value	Cohen’s *d*	CI Lower (95%)	CI Upper (95%)
Vehicle	9	0.77	0.10	_				
MGO 10 μM	9	1.46	0.21	vs. vehicle	<0.0001	4.19	2.54	5.84
MGO + HC-030031	6	0.77	0.25	vs. MGO 10μM	<0.0001	−3.05	−4.55	−1.54
MGO + HC-067047	7	0.98	0.42	vs. MGO 10μM	0.0032	−1.51	−2.63	−0.39
	**pEC_50_**							
Vehicle	9	6	0.09	_				
MGO 10 μM	9	5.98	0.18	vs. Vehicle	0.99	−0.4	−1.06	0.78
MGO + HC-030031	6	6.01	0.24	vs. MGO 10 μM	0.99	0.14	−0.88	1.18
MGO + HC-067047	7	5.47	0.42	vs. MGO 10 μM	0.001	−1.66	−2.8	−0.51

Data are expressed as mean ± SD. Effect size was calculated as Cohen’s *d*, accompanied by 95% confidence intervals (CIs). One-way ANOVA followed by Dunnett’s multiple comparisons test versus vehicle groups.

**Table 5:** Values of Emax and potency (pEC_50_) of carbachol-induced contractions in intact and denuded bladder strips incubated with MGO (300 μM) in the presence or not of L-NAME (100 μM).

Emax (mN/mg)									
	Group	*n*	Mean	SD	Comparison	*p*-Value	Cohen’s *d*	CI Lower (95%)	CI Upper (95%)
**Intact**	MGO (untreated)	6	1.04	0.37	__				
	MGO + L-NAME	5	1.78	0.5	vs. untreated	0.02	1.71	0.32	3.09
**Denuded**	MGO (untreated)	17	0.85	0.34	__				
	MGO + L-NAME	9	1.85	0.51	vs. untreated	<0.0001	2.47	1.42	3.52
**pEC_50_**									
**Intact**	MGO (untreated)	6	5.82	0.32	__				
	MGO + L-NAME	5	5.91	0.2	vs. untreated	0.59	0.32	−0.86	1.52
**Denuded**	MGO (untreated)	17	5.75	0.37	__				
	MGO + L-NAME	9	5.66	0.21	vs. untreated	0.50	−0.27	−1.08	0.53

Data are expressed as mean ± SD. Effect size was calculated as Cohen’s *d*, accompanied by 95% confidence intervals (CIs). One-way ANOVA followed by Dunnett’s multiple comparisons test versus vehicle groups.

**Table 6:** Values of Emax and potency (pEC_50_) of carbachol-induced bladder contractions in triple knockout (KO) mice for nitric oxide synthase (NOS; neuronal/endothelial/inducible NOS^−/−^) incubated with MGO (300 μM).

Emax (mN/mg)								
Group	*n*	Mean	SD	Comparison	*p*-Value	Cohen’s *d*	CI Lower (95%)	CI Upper (95%)
Vehicle	4	0.61	0.29	__				
MGO	4	1.11	0.27	vs. vehicle	0.04	1.75	0.12	3.38
**pEC_50_**								
Vehicle	4	5.82	0.26	__				
MGO	4	5.58	0.20	vs. vehicle	0.19	−1.03	−2.51	0.44

Data are expressed as mean ± SD. Effect size was calculated as Cohen’s *d*, accompanied by 95% confidence intervals (CIs). One-way ANOVA followed by Dunnett’s multiple comparisons test versus vehicle groups.

**Table 7:** Values of Emax and potency (pEC_50_) of carbachol-induced bladder contractions in endothelial nitric oxide synthase (eNOS) knockout mice (eNOS^−/−^) incubated with MGO (300 μM).

	Emax (mN/mg)							
Group	*n*	Mean	SD	Comparison	*p*-Value	Cohen’s *d*	CI Lower (95%)	CI Upper (95%)
Vehicle	4	0.69	0.23	__				
MGO	4	1.11	0.16	vs. vehicle	0.02	2.12	0.38	3.85
**pEC_50_**								
Vehicle	4	5.35	0.58	__				
MGO	4	5.48	0.28	vs. vehicle	0.70	0.28	−1.1	1.67

Data are expressed as mean ± SD. Effect size was calculated as Cohen’s *d*, accompanied by 95% confidence intervals (CIs). One-way ANOVA followed by Dunnett’s multiple comparisons test versus vehicle groups.

**Table 8:** Glyoxalase 1 (Glo1) activity in bladder tissues incubated with varying concentrations of MGO.

Concentration	Glo1 Activity in Bladder (IU/mg Protein)	*p*-Value
Vehicle	0.50 ± 0.05	
MGO 10 μM	0.58 ± 0.10	0.99
MGO 30 μM	0.53 ± 0.11	0.37
MGO 100 μM	0.50 ± 0.11	0.92
MGO 300 μM	0.50 ± 0.06	0.99

Data are presented as mean ± SD (*n* = 7 animals per group). One-way ANOVA followed by Dunnett’s multiple comparisons test versus the vehicle group. Vehicle: saline.

## Data Availability

The original metadata and data presented in the study are openly available in the UNICAMP Research Data Repository—REDU, at: https://doi.org/10.25824/redu/VYNGJ7.

## Abbreviations

The following abbreviations are used in this manuscript:

ACh	Acetylcholine
AGEs	Advanced glycation end products
DBD	Diabetic bladder dysfunction
DM	Diabetes mellitus
EFS	Electrical field stimulation
eNOS	Endothelial nitric oxide
Glo1	Glyoxalase 1
MGO	Methylglyoxal
NO	Nitric oxide
PEG-SOD	Polyethyleneglycol-superoxide dismutase
RAGE	Receptor of AGEs
RAP	RAGE antagonist peptide
ROS	Reactive oxygen species
SOD	Superoxide dismutase
TRPA1	Transient receptor potential ankyrin 1
TRPV4	Transient receptor potential vanilloid 4
